# Danggui Buxue Tang Rescues Folliculogenesis and Ovarian Cell Apoptosis in Rats with Premature Ovarian Insufficiency

**DOI:** 10.1155/2021/6614302

**Published:** 2021-05-05

**Authors:** Lingdi Wang, Jian Liu, Guangning Nie, Yang Li, Hongyan Yang

**Affiliations:** ^1^Guangzhou University of Chinese Medicine, Guangzhou 510006, Guangdong, China; ^2^Department of Gynaecology, The Second Affiliated Hospital of Guangzhou University of Chinese Medicine, Dade Road, Yuexiu District, Guangzhou 510120, Guangdong, China; ^3^Guangdong Provincial Key Laboratory of Clinical Research on Traditional Chinese Medicine Syndrome, Guangzhou, Guangdong, China

## Abstract

Premature ovarian insufficiency (POI) is a common female endocrine disease that is closely linked to ovarian function. Danggui Buxue Tang (DBT) is a classic prescription of traditional Chinese medicine that is helpful for rescuing ovarian function. However, the mechanism by which DBT rescues ovarian function remains unclear. This study explored the molecular mechanism of DBT with respect to apoptosis and related signals in ovarian cells. The quality control of DBT was performed by HPLC. After DBT intervention in the POI rat model, serum AMH/FSH/LH/E_2_ levels were detected by ELISA, follicles at various developmental stages were observed by HE staining, apoptosis was detected by TUNEL, and the expression profiles of Bcl-2 family proteins and key proteins in the Jak2/Foxo3a signaling pathway were evaluated by western blot. The results demonstrated that DBT could encourage the development of primary/secondary/antral follicles and increase the secretion of AMH. Moreover, DBT might inhibit Foxo3a by upregulating Jak2, thereby mediating Bcl-2 family activities and inhibiting apoptosis in ovarian cells. In conclusion, DBT promotes follicular development and rescues ovarian function by regulating Bcl-2 family proteins to inhibit cell apoptosis, which could be related to the Jak2/Foxo3a signaling pathway.

## 1. Introduction

Premature ovarian insufficiency (POI) refers to the occurrence of menopause before the age of 40, which can cause a series of diseases of the reproductive system, cardiovascular system, osteoporosis, and related conditions [1, 2]. The etiology of POI is not yet known. Common causes include chromosomal abnormalities or gene mutations, immunity, viral infections, and iatrogenic factors [3]. Approximately 70–80% of cases of POI have an unclear cause and are also known as idiopathic premature ovarian insufficiency.

One of the most notable characteristics of ovarian aging is the gradual loss of the primordial follicle pool [4], and metabolic products and oxidative stress have damaging effects on mitochondrial function and the development of oocytes. In addition, granulosa cell apoptosis increases, which can aggravate follicle damage [5]. Follicular atresia is closely related to oocyte and granulosa cell apoptosis. Ovarian granulosa cells surround the oocyte and provide hormones, regulatory factors, and signal factors for the development of follicles [6]. Abnormal apoptosis of oocytes or granulosa cells triggers follicle exhaustion, leading to ovarian aging [7]. Studies have shown that ovarian failure is related to apoptosis [8], which might be associated with oxidative stress [9], DNA damage [10, 11], autophagy [12], and related pathways. Apoptosis plays an important role in vertebrates and is regulated by the interaction of three subgroups of Bcl-2 family proteins on the outer membrane of mitochondria: proapoptotic proteins, antiapoptotic proteins, and BH3-only proteins [13]. These molecular signals also influence each other in a complicated network, and the mechanism of POI has not yet been clarified.

Hormone replacement therapy (HRT) is utilized to ameliorate menopausal symptoms in many menopausal women. However, HRT is known to cause breast cancer, ovarian cancer, uterine cancer, and gallbladder diseases; most important of all, HRT cannot reconstruct ovarian function [14]. Thus, the exploration of useful drugs to restore ovarian function is still a focus. Complementary and alternative treatments in Chinese medicine have been recognized worldwide. In China, traditional Chinese medicines (TCMs) have served as medicines or health supplements over thousands of years. Danggui Buxue Tang (DBT, an herbal decoction) is a simple combination of two herbs, Astragali Radix (AR) and Angelicae Sinensis Radix (ASR), in a weight ratio of 5 : 1. Traditionally, DBT is prescribed for women in China as a remedy for menopausal symptoms and can improve their health by raising the “Qi” (vital energy) and nourishing the “Blood” (body circulation). Today, DBT is used in various herbal prescriptions for treating menopausal symptoms. Recent studies have revealed the pharmacological properties of DBT both in vivo and in vitro. Studies have shown that DBT can promote megakaryocyte proliferation and inhibit apoptosis to promote hematopoiesis and plateletogenesis [15]. DBT can induce the proliferation of artificial hematopoietic stem cells and reduce their apoptosis by inhibiting Fas-mediated apoptosis and activation of the JAK signal transduction pathway induced by interferon regulatory factors [16]. In addition, DBT has been demonstrated to have anti-inflammatory effects and can treat renal fibrosis by inhibiting inflammatory factors [17]. However, the potential mechanism by which DBT saves ovarian function is unclear. This study aimed to explain the apoptosis-related molecular mechanism underlying the effect of DBT in POI treatment.

## 2. Materials and Methods

The experiment passed the ethical review of the Experimental Animal Ethics Committee of Guangdong Provincial Hospital of Traditional Chinese Medicine.

### 2.1. Chemicals and Reagents

4-Vinylcyclohexene diepoxide (VCD) (BCBT2645) was purchased from Sigma-Aldrich, Korea. ELISA kits were purchased from Wuhan Huamei Biological Engineering Company, Wuhan, China. Antibodies against Bcl-2 (2876 s), Bax (2772 s), Cleaved-caspase3 (14220 T), and Jak2 (3230) were purchased from Cell Signaling Technology, USA. Antibodies against Bim (ab17935) and Bcl-xl (ab32370) were purchased from Abcam (Shanghai, China). The antibody against Foxo3a (10849-1-AP) was purchased from Proteintech (Wuhan, China). A TUNEL assay kit (11684817910) was acquired from Roche, Shanghai, China.

### 2.2. Herbal Materials and DBT Extract

*Astragalus* (180,403,651) and *Angelica* (1,804,001) were purchased from Kangmei Pharmaceutical Company, Guangdong, China. By identifying the chemical and biological properties of DBT (*Astragalus* and *Angelica*, at a ratio of 5 : 1), the optimized conditions for herbal extraction were established. DBT was extracted with pure water. After being soaked for 30 minutes, DBT was then boiled in a condensing reflux device. Heating was continued for 60 minutes after boiling. The extraction operation was replicated twice, and then all the mixed liquid was collected. The mixed liquid was concentrated to 1 mg/ml with a rotary evaporator and stored at −20°C for subsequent use.

### 2.3. Quality Control and High-Performance Liquid Chromatography (HPLC)

For evaluation of the quality consistency of DBT extracts and batch-to-batch consistency studies, three chemical standards were utilized as markers, namely, calycosin-7-glucoside, formononetin, and ferulic acid. All these standard chemicals were purchased from Shanghai Yuanye Biotechnology Co., Ltd. (Shanghai, China). Briefly, three batches of DBT extracts were accurately weighed out and extracted. The solution was filtered through a 0.22-*μ*M syringe filter and the filtrate was invoked as the test solution. Samples were analyzed on a Diamonsil C18 column (250 mm × 4.6 mm I. D, 5 *μ*m; Dikma Technologies Inc.) in an Agilent 1200 system (Agilent Technologies, Inc., Santa Clara, CA, USA) with a VWD detector at 290 nm. The mobile phase consisted of 0.1% formic acid aqueous solution (A) and methanol (B) using gradient elution (0 min: 5% B, 10 min: 25% B, 15 min: 35% B, 30 min: 38% B, 35 min: 50% B, 45 min: 65% B, 55 min: 90% B) at a flow rate of 1.0 ml/min, and the injection volume was 10 *µ*l. Reproducibility and linearity were estimated.

### 2.4. POI Model Establishment and Groups

The animals used throughout this study were SPF-level SD female rats (28 days of age) provided by Guangdong Medical Laboratory Animal Center (license number: 44007200053829). Fifteen female SD rats were randomly divided into 3 groups: the control group (CON group), the VCD-induced model group (VCD group), and the VCD + DBT group (DBT group). The VCD-induced SD rat model has been reported previously [16]. Both VCD-induced rats and DBT-treated rats received VCD (160 mg/kg) intraperitoneal injections daily for 15 days. After the last VCD intraperitoneal injection, the rats in the VCD + DBT group received a 7.5 g/kg/day dose of DBT for 75 consecutive days. The dosage and duration of VCD and DBT were determined according to a previous article [18].

### 2.5. Estrous Cycle

Vaginal secretion smears were collected at 8–10 am every day 30 days after the last VCD treatment day. The normal estrous cycle was defined for 4 or 5 days of regular changes as a turn of proestrous, estrous, postestrous, and estrus intervals. Any pattern that did not follow the normal cycle was considered a disorder [19].

### 2.6. Tissue Collection and Preparation

After the rats were properly anesthetized, blood was taken from the abdominal aorta. Then, the ovaries, uterus, and fallopian tubes were excised separately after the last day of the treatment. A total of 30 ovaries, 15 uteri, and 30 fallopian tubes were obtained. After tissues were excised from animals, each ovary or uterus was weighed on an electronic balance. The ovaries on either side of each rat and all uterine and fallopian tubes were soaked in 4% paraformaldehyde and were then used to prepare paraffin sections for HE staining or TUNEL assay. The remaining ovaries were fully lysed by RIPA buffer to extract protein for western blot analysis. At the same time, serum from all rats was obtained to test related hormone levels using an ELISA kit.

After fixing the tissue with 4% paraformaldehyde, the tissue was washed with running water. Then, tissues were dehydrated in gradient ethanol (concentrations of 50%, 60%, 70%, 80%, 90%, and 95%). The cells were treated with xylene for transparency and were then embedded in paraffin. Tissue sections were cut into 5-*μ*m-thick sections by an automatic slicer, and one slice was picked out every 40 slices. Approximately 8–10 slices were taken from one ovary for follicular morphology observation and follicle counting after HE staining. In addition, one slice was removed every 20 slices, and approximately 5 slices were derived from one ovary for subsequent TUNEL staining.

### 2.7. HE Staining and Follicle Classification

The paraffin slices were soaked in xylene, soaked in ethanol solutions of different concentrations for dehydration, and then washed with running water. Cell nuclei were stained with hematoxylin and washed with water until they ran clear. Sections were soaked in 1% hydrochloric acid alcohol for differentiation. The cytoplasm was stained with eosin and washed with water. A gradient concentration of ethanol was used for dehydration, and xylene was used for transparency. Neutral gum was used to seal the slices. After HE staining, the ovarian sections were observed, and the follicles were counted under a 400x optical microscope.

Follicles were divided into the following types by existing standards [20]: primordial follicles were surrounded by several granular cells. Primary follicles were surrounded by a single layer of cubic granular cells. Secondary follicles were surrounded by multiple granular cells. The antral follicle possessed a follicular cavity full of follicular fluid. Mature follicles possessed a larger follicular cavity, with cumulus cells surrounding the oocyte. Atretic follicle walls collapsed, the structures were not clear or even disappeared, and the zona pellucida shrank.

### 2.8. TdT-Mediated dUTP Nick-End Labeling (TUNEL) Assay

The paraffin sections were deparaffinized and hydrated, and 0.1% Triton X-100 and 0.1% sodium citrate were added to increase permeability. A mixture of 3% BSA and 20% normal calf serum was used to prevent nonspecific reactions. The serum was shaken off, and the TUNEL reaction mixture was added dropwise to each section and incubated for 60 minutes. The working solution was replaced with TBS in the negative control group. Next, 3% H_2_O_2_ was added for blocking, and the cells were stored in the dark at room temperature for 10 minutes. The sections were then blocked again with 20% goat serum. POD solution was added and incubated for 30 minutes. Freshly prepared DAB solution was added for color development, and the color development time was controlled under a microscope to halt color development. The cells were stained with hematoxylin for 1 minute, differentiated in hydrochloric acid alcohol for 1 to 2 seconds, and turned blue with blue liquid. The sections were dehydrated and dried in gradient alcohol, transparentized by xylene, and finally sealed by neutral gum for microscopic observation.

### 2.9. Western Blot Analysis

The proteins were extracted from ovaries obtained from the CON group, the VCD group, and the DBT group and were boiled with proper loading buffer for 10 minutes. Protein concentration was measured by BCA assay. Fifty micrograms of protein from each group was separated by 12% SDS-PAGE and then transferred onto a nitrocellulose filter membrane. The membranes were blocked with 5% BSA for 1.5 h at room temperature and then incubated with the appropriate primary antibody at 4°C overnight. The next day, the membranes were washed with TBST 3 times (10 minutes each time). The membranes were incubated with the corresponding secondary antibodies for one hour. After 3 washes with TBST, blot images were obtained with a Bio-Rad ChemiDoc XRS + chemiluminescence imaging system. The results were analyzed by ImageJ software.

### 2.10. Statistical Analysis

All data were analyzed with SPSS 24.0 software. The data conformed to a normal distribution and homogeneity of variance. Data satisfying a normal distribution are expressed as the mean ± standard deviation. Data that did not meet the normal distribution are expressed as percentiles. When the data were satisfied with a normal distribution and homogeneity of variance, one-way ANOVA was used to compare the differences within 3 groups, and LSD was used to compare every two groups. When the data did not satisfy the normal distribution, Kruskal-Wallis tests were used to compare the differences within 3 groups, and Bonferroni tests were used to compare every two groups. When the data variance was not homogeneous, Welch's test was used to analyze the difference within 3 groups, and the Games Howell post hoc test was used to compare every two groups. *P* < 0.05 indicated a significant difference.

## 3. Results

### 3.1. Determination and Analysis of Three Compositions in the DBT Aqueous Extract Solution

**Figure 1:**
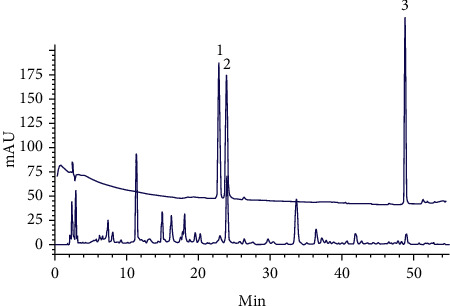
Identiﬁcation and quantiﬁcation of campanulin, ferulic acid and formononetin in DBT by HPLC analysis (1: campanulin, 2: ferulic acid, 3: formononetin). Mobile phase A was an 8.5% formic acid aqueous solution, and mobile phase B was formic acid/acetonitrile/methanol/water (8.5 : 22.5 : 22.5 : 41.5, *v*/*v*/*v*/*v*). The linear gradient program was as follows: 0–35 min from 7 to 25% B, 35–45 min from 25 to 65% B, 45–46 min from 65 to 100% B, and 46–50 min 100% B. The ﬂow rate was 1.0 mL/min, and the absorption spectrum was recorded at 535 nm. The column was operated at a temperature of 30°C. Three replicates were performed in the analysis.

A chromatographic fingerprint of DBT was characterized via HPLC under the optimum running conditions using three standard compounds, and three batches of DBT preparations were examined. The obtained chromatograms are given in Figure 1. By considering the amounts of the three standard compounds in DBT, a calibration curve was constructed with six concentration levels, and the correlation coefficient values of the calibration curves were over 0.997. HPLC analysis showed that the contents of campanulin, ferulic acid, and formononetin in DBT were 0.330 mg/g, 0.882 mg/g, and 0.407 mg/g, respectively. (Table 1).

### 3.2. Effects of DBT on Body Weight and Organ Coefficients of the Ovary and Uterus

**Figure 2:**
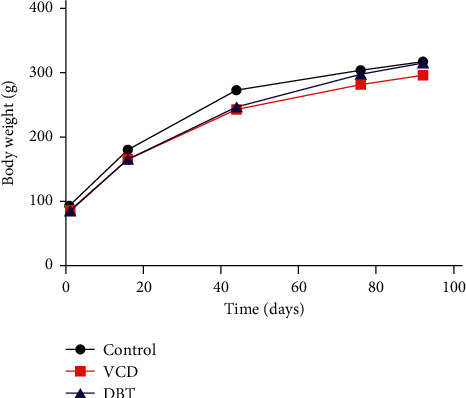
Changes in the weight of rats during the first to fifth months of the experiment.

To determine the effects of DBT on the ovaries and uteri of rats, the weights of the ovaries and uteri were measured at the end of the experiment. The weight of the rat was measured monthly during the experiment. At the end of treatment, all of the rats were alive, and no significant differences were found in the body weights of the rats in the various groups (*P* > 0.05, Figure 2). The organ coefficients were calculated with the final weight at the end of the experiment. Both ovarian weight and the ovarian organ coefficient in the VCD group were decreased compared with those in the CON group (*P* < 0.05). The ovarian organ coefficient was increased in the DBT group compared with the VCD group (*P* < 0.05). No significant differences in uterine weight were observed among the groups. The results are summarized in Table 2.

### 3.3. Observation of the Estrous Cycle of Rats

**Figure 3:**
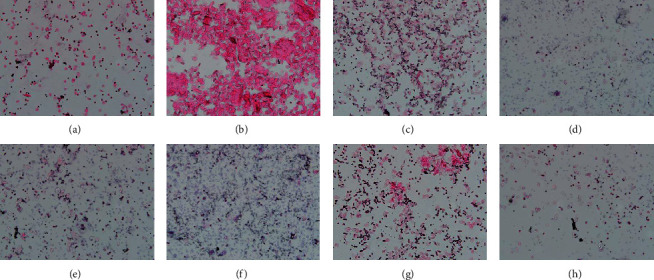
Regular and irregular estrous cycle patterns in rats. Regular estrous cycle in the CON or HYF group (from (a) to (d) HE, ×40) and irregular estrous cycle in the VCD group (from (e) to (h) HE, ×40).

The estrous cycle of the rats was observed during the experiment. All rats in the CON group possessed regular estrous cycles, and all rats in the VCD group lost normal estrous cycle patterns. In the DBT group, 60% of rats resumed regular estrous cycles within 7 days before sacrifice. The regular and irregular estrous cycle patterns in rats are displayed in Figure 3.

### 3.4. Effects of DBT on Serum Sex Hormone Levels

Sex hormone levels reflect ovarian function, especially AMH. To observe the effect of DBT on ovarian reproductive endocrine function, the levels of sex hormones in rats were measured. As shown in Table 3, AMH levels were increased in the DBT group compared with the VCD group (*P* < 0.05), and no statistically significant changes were observed among the groups in E_2_/FSH/LH.

### 3.5. Effects of DBT on Ovarian Follicular Reserve and Development

**Figure 4:**
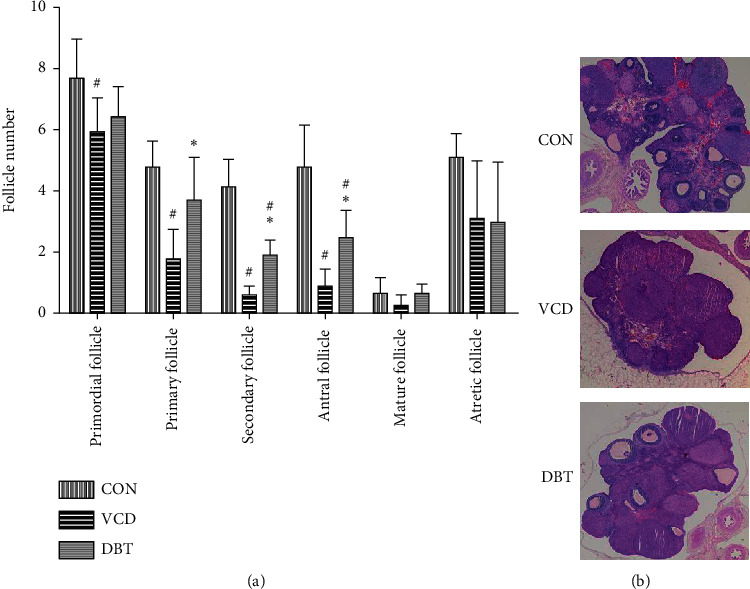
Effect of DBT on follicle development in the ovary. (a) Follicle counts in major developmental stages; (b) appearance of ovaries in the three groups by HE staining. ^#^*P* < 0.05, compared with the CON group; ^*∗*^*P* < 0.05, compared with the VCD group.

To assess whether DBT affects follicular development, HE-stained ovarian paraffin sections were used to count follicles at various developmental stages. Five developmental types of follicles in the three groups were counted. In histopathologically stained sections of rat ovaries from the CON group, large numbers of visible, mature follicles were seen at various stages of development (Figure 3(b)). After 15 days of daily dosing, VCD induced significant losses of primordial follicles, primary follicles, secondary follicles, and antral follicles compared with the CON group (*P* < 0.05). In ovarian sections from POF rats treated with DBT, mature follicles resembling those in control rats were observed, while, compared with the VCD group, the numbers of primary, secondary, and antral follicles in the DBT group were increased (*P* < 0.05). The results are shown in Figure 4.

### 3.6. Effect of DBT on Apoptosis of Ovarian Cells

**Figure 5:**
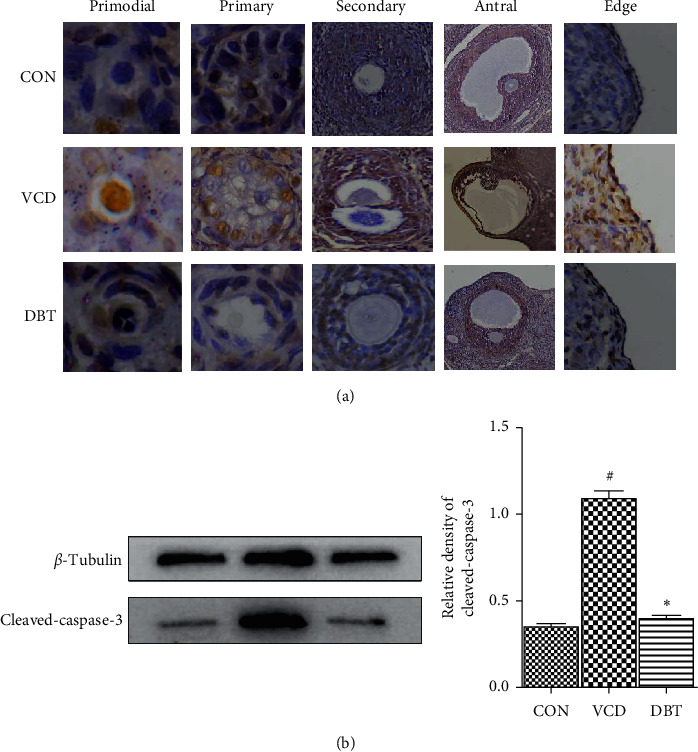
Effect of DBT on the apoptosis of ovarian cells. (a) TUNEL staining in the ovaries and the ovarian follicles in the three groups. The apoptotic cells on the tissue section under the microscope are brown. Scale bars = 100 *μ*m (primordial, primary); 50 *μ*m (secondary, edge); 10 *μ*m (antral). (b) The apoptosis executive protein cleaved caspase-3 was tested by western blotting. ^#^*P* < 0.05, compared with the CON group; ^*∗*^*P* < 0.05, compared with the VCD group.

Ovarian cell apoptosis is a natural process, and there is a balance between proliferation and apoptosis, which affects normal ovarian function. To determine the effect of DBT on ovarian apoptosis, ovarian sections from the three groups were tested by the TUNEL assay. The TUNEL assay easily distinguished between apoptotic granulosa cells (TUNEL-positive cells) and others. As shown in Figure 5, apoptosis changes in the ovary and developing follicles were captured. A large number of apoptotic primordial follicle oocytes and primordial follicle granulosa cells were detected in the VCD groups; a very small amount of positive staining was seen in primordial follicle granulosa cells and primordial follicle oocytes in the CON or DBT groups. TUNEL positivity was captured in primary follicles or secondary follicle oocytes and granulosa cells in the three groups; however, in primary follicles or secondary follicles and granulosa cells of VCD-treated rats, widespread TUNEL positivity was observed. Overall, apoptosis in the VCD group was increased, and in the DBT group, apoptosis was extensively rescued. This is consistent with the analysis of follicular count results. In addition, to verify the apoptotic results of the TUNEL assay, we detected the protein expression of cleaved caspase-3, which is recognized as the initial executor of the apoptosis process. The expression of cleaved caspase-3 was significantly increased in the VCD group (*P* < 0.05) and was recovered in the DBT group (*P* < 0.05).

### 3.7. The Effect of DBT on Ovarian Apoptosis Was Related to the Bcl-2 Family and Jak2/Foxo3a Signaling

**Figure 6:**
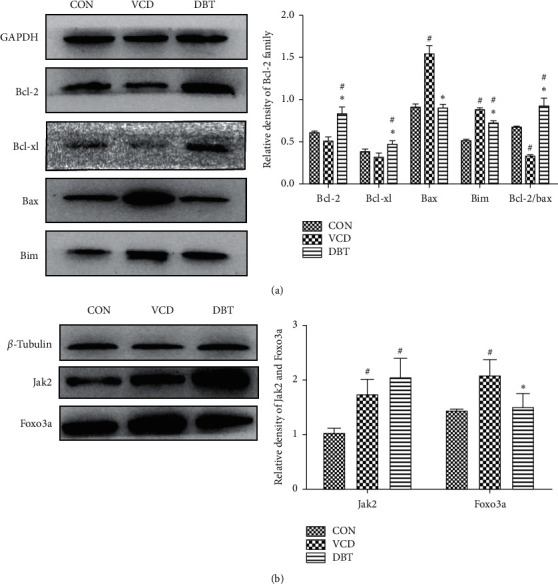
The relationship between DBT conservation of the ovary and the Bcl-2 family and Jak2/Foxo3a signaling pathway. ^#^*P* < 0.05, compared with the CON group; ^*∗*^*P* < 0.05, compared with the VCD group.

**Figure 7:**
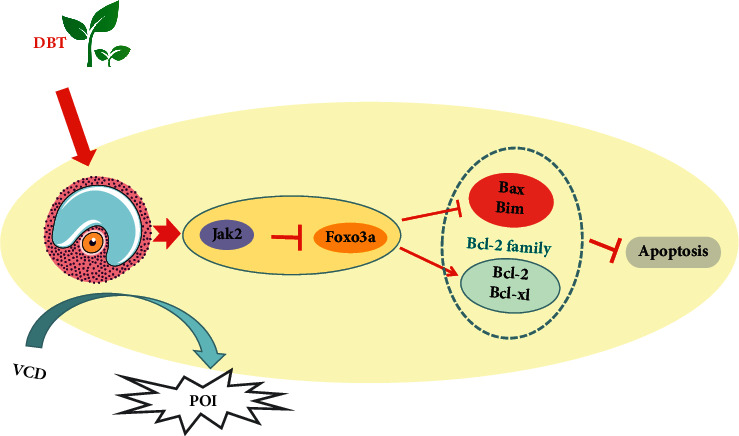
Schematic diagram of DBT regulating apoptosis in POI rats.

Bcl-2 family proteins were verified by western blot. As displayed in Figure 6(a), the expression levels of proapoptotic proteins (Bax and Bim) and antiapoptotic proteins (Bcl-2 and Bcl-xl) showed specific variations: the expression levels of Bcl-2 and Bcl-xl decreased in the VCD group and increased in the DBT group, while the expression of Bim increased in the VCD group and increased in the DBT group. Additionally, the expression of Bax did not show apparent changes among the three groups. The age-related JAK and FOXO signaling pathways were reported to be possibly involved in oocyte development [21] and cellular senescence [22]. Thus, the main factors Jak2 and Foxo3a were verified by western blot. As shown in Figure 6(b), Jak2 and Foxo3a were both decreased in the VCD group, while compared with the VCD group, the expression of Jak2 was increased and the expression of Foxo3a was decreased. According to the present results, VCD could regulate Bcl-2 family proteins by increasing Foxo3a, leading to an increase in apoptosis; in addition, DBT might inhibit Foxo3a by the upregulation of Jak2, which mediated the expression of antiapoptotic proteins and suppressed proapoptotic proteins, ultimately reducing apoptosis. The mechanism is displayed in Figure 7.

## 4. Discussion

Chinese medicine has been clinically proven to have an effect on treating female ovarian hypofunction [23]. DBT is a well-known Chinese medicine prescription that is widely recognized for improving blood deficiency diseases. DBT has been reported to promote erythropoietin, platelets, and angiogenesis [23]. The effects of stimulating the immune response [24] and alleviating myocardial damage [25] have also been reported [24]. DBT is composed of Angelica and *Astragalus*, and both herbs showed possible improvement in ovarian function. Ferulic acid in Angelica has antioxidant effects and could reduce the damage of ROS to ovarian function [26]. Ferulic acid was also reported to increase the number of larger follicles in mammalian ovaries [27]. *Astragalus* has been reported to have anti-inflammatory [28] and antioxidant effects [29] and could promote spermatogenesis in KM mice [30]. However, there are few studies on the mechanism by which DBT preserves ovarian reproductive function.

Overall, ovarian reserve depletion “burn out,” and direct oocyte damage are the major reasons for POI patients to have no follicles available, problems that have not been resolved to date. In this study, we aimed to explore whether DBT exerts the clinical effect of treating POI through apoptosis-related signals. POI usually shows fluctuations in sex hormones, including a decrease in AMH and E_2_ and an increase in FSH [31]. The main effect is to hinder the development of follicles, causing infertility [32]. Based on previous studies, we selected VCD intraperitoneal injection as the POI model for this study [33]. Our results showed that DBT did not seem to have significant effects on FSH, E_2,_ or LH levels, but compared with the model group, the decrease in AMH was recovered in the DBT group. This result implies that DBT has an effect on the restoration of AMH, which is the most important and sensitive response to ovarian function [34]. In addition, we counted the number of follicles at several stages in the three groups. Significant changes occurred in the primary, secondary, and antral follicular stages. DBT obviously saves the primary, secondary, and antral follicles that are damaged by VCD, helping to restore follicle development. In addition, we found that the VCD model also reduced the number of primordial follicles, but DBT has not yet shown a significant effect on the restoration of primordial follicles. This result suggests that the recovery of primordial follicles is difficult, as reported before [35]. Preantral follicles and small antral follicles are the main follicular stage for secreting AMH [35]. In our study, the rescue of antral follicles in the DBT group was consistent with the increase in AMH levels compared with the VCD group. All the above results show that DBT can improve ovarian reproductive function by increasing the level of AMH and the numbers of primary/secondary follicles and antral follicles.

To determine the effect of DBT on the apoptosis of ovarian cells, the apoptosis of the three groups was detected by TUNEL assay. The VCD group showed obvious apoptotic cells at every follicular stage, while the DBT group had less apoptosis than the VCD group. In addition, since caspase-3 is recognized as the initial executor of the apoptosis process [36], the significant changes in the three groups were verified by western blot. Consistent with a previous study [36], the western blot results in this study also showed that apoptosis was clearly increased in the VCD group, and our study showed that DBT could reverse this detrimental increase. The above verification suggests that DBT works by inhibiting ovarian cell apoptosis.

Furthermore, with the purpose of exploring the molecular mechanism of DBT intervention in ovarian cell apoptosis, we next detected Bcl-2 family proteins, which are reported as apoptosis-related signals [37]. The mitochondrial signaling pathway is one of the major signaling pathways that conduct cell apoptosis [38], and the main controller of the mitochondrial signaling pathway is the Bcl-2 family [39, 40]. The Bcl-2 family is mainly divided into two categories in terms of function: proapoptotic proteins (Bcl-2, Bcl-xl, etc.) and antiapoptotic proteins (Bax, Bak, Bim, Bad, etc.) [39, 40]. As previously reported [13], Bcl-2 and Bax are a pair of antagonistic partners and play opposite roles in the mitochondrial apoptosis pathway. The ratio of Bcl-2/Bax could reflect cell proliferation and apoptosis [41]. In our study, Bcl-2, Bcl-xl, Bax, and Bim were detected by western blot, and the results showed that both Bcl-2 and Bcl-xl were decreased in the VCD group but were rescued in the DBT group. In addition, the expression of Bax did not change evidently, as previously reported [6], though the expression of Bim and the Bcl-2/Bax ratio indicated significant variations between the VCD and DBT groups, consistent with previous reports [42]. The above evidence suggests that DBT regulates ovarian cell apoptosis by affecting Bcl-2 family proteins.

According to a previous study, transcriptomics analysis of differential gene expression found that differentially expressed genes in ovarian follicle activation include the Jak and Foxo signaling pathways [42]. It is worth mentioning that Foxo3a was strongly related to follicular development and fate: on the one hand, Foxo3a can inhibit the excessive activation of primordial follicles and maintain the normal reserve of the follicular pool [43]; on the other hand, follicular development was retarded, and granulosa cell proliferation was arrested with Foxo3a-specific expression [44]. Another study showed that in POI patient ovaries, the expression of Foxo3a was decreased [45]. Foxo3a is likely to regulate follicle fate by triggering Bcl-2 family members such as Bim and Bax [46]. In our early report, the Jak2/Stat signaling pathway might play a potential role in the recovery of ovarian function [18]. In this study, Foxo3a and Jak2 were selected to examine whether they have an effect on the molecular mechanism of DBT in remediating ovary function. Our results show that the significant increases in Jak2 and Foxo3a indicate that they are both involved in ovarian apoptosis. Interestingly, Jak2 expression was upregulated again in the DBT group, and Foxo3a expression was reduced. Consistent with a previous report [47], the level of Foxo3a was negatively correlated with the activation of Jak2. Based on the previous reports mentioned above, VCD may damage ovarian cells by affecting the expression of Foxo3a signals, and DBT could carry out a rescue function through the Jak2/Foxo3a pathway.

## 5. Conclusion

In summary, the present study suggests the potential molecular mechanism of DBT in the treatment of POI. We found that DBT rescues ovarian function by promoting the development of primary/secondary/antral follicles and increasing the secretion of AMH. Furthermore, DBT alleviated abnormal ovarian apoptosis through the Jak2/Foxo3a pathway by inhibiting the expression of proapoptotic proteins and promoting the expression of antiapoptotic proteins in the Bcl-2 family. This study is a preliminary exploration of DBT to rescue ovarian function. Further studies will provide a broad scientific basis for DBT to protect female reproductive endocrine function.

## Figures and Tables

**Table 1:** Contents of three compound standards in DBT.

Standards (*µ*g/mg)	Retention time (min)	Batch 1	Batch 2	Batch 3	Mean	Standard deviation
Campanulin	22.9	0.373	0.298	0.316	0.330	0.040
Ferulic acid	23.9	0.907	0.846	0.894	0.882	0.032
Formononetin	49.0	0.437	0.378	0.405	0.407	0.030

**Table 2:** Body weight, ovarian/uterine weight, and ovarian/uterine organ coefficient of each group of rats.

Groups	Body weight (g)	Bilateral ovarian weight (mg)	Ovarian organ coefficient (%)	Uterine weight (mg)	Uterine organ coefficient (%)
CON	316.8 ± 27.96	80.0 (78.6∼94.4)	0.027 ± 0.003	472.00 ± 79.12	1.502 ± 0.263
VCD	315.8 ± 34.75	62.6 (60.4∼63.4)^△^	0.022 ± 0.003^#^	513.82 ± 54.62	1.649 ± 0.251
DBT	312.8 ± 9.06	80.2 (79.6∼82.4)	0.026 ± 0.004	510.24 ± 87.92	1.636 ± 0.269

^Δ^ *P* < 0.05, compared with the CON group; ^#^*P* < 0.05, compared with the CON group.

**Table 3:** Changes in serum sex hormones in rats in each group.

Groups	AMH (ng/ml)	E2 (pg/ml)	FSH (mIU/ml)	LH (pg/ml)
CON	25.54 ± 5.56	63.48 ± 9.53	16.14 (15.04∼18.36)	3.86 ± 0.47
VCD	15.37 ± 0.49^#^	58.53 ± 4.34	12.58 (12.22∼17.09)	3.97 ± 0.38
DBT	20.73 ± 1.83^*∗*^	60.65 ± 4.39	14.10 (12.44∼18.00)	4.08 ± 0.33

^#^ *P* < 0.05, compared with the CON group; ^*∗*^*P* < 0.05, compared with the VCD group.

## Data Availability

The data used to support the findings of this study are available from the corresponding author upon request.
